# Signaling pathways governing the maintenance of breast cancer stem cells and their therapeutic implications

**DOI:** 10.3389/fcell.2023.1221175

**Published:** 2023-07-10

**Authors:** Alejandro Ordaz-Ramos, Olivia Tellez-Jimenez, Karla Vazquez-Santillan

**Affiliations:** ^1^ Innovation in Precision Medicine Laboratory, Instituto Nacional de Medicina Genómica, Mexico City, México; ^2^ Posgrado en Ciencias Biológicas, Unidad de Posgrado, Circuito de Posgrados, Ciudad Universitaria, Coyoacán, México

**Keywords:** cancer stem cells, pathways, self-renewal, breast cancer, therapy

## Abstract

Breast cancer stem cells (BCSCs) represent a distinct subpopulation of cells with the ability to self-renewal and differentiate into phenotypically diverse tumor cells. The involvement of CSC in treatment resistance and cancer recurrence has been well established. Numerous studies have provided compelling evidence that the self-renewal ability of cancer stem cells is tightly regulated by specific signaling pathways, which exert critical roles to maintain an undifferentiated phenotype and prevent the differentiation of CSCs. Signaling pathways such as Wnt/β-catenin, NF-κB, Notch, Hedgehog, TGF-β, and Hippo have been implicated in the promotion of self-renewal of many normal and cancer stem cells. Given the pivotal role of BCSCs in driving breast cancer aggressiveness, targeting self-renewal signaling pathways holds promise as a viable therapeutic strategy for combating this disease. In this review, we will discuss the main signaling pathways involved in the maintenance of the self-renewal ability of BCSC, while also highlighting current strategies employed to disrupt the signaling molecules associated with stemness.

## 1 Introduction

Inside tumor mass, a distinct subset of malignant cells possesses the remarkable ability to establish the intra-tumor heterogeneity and sustain tumor growth and metastasis. Such cells, defined as cancer stem cells (CSCs), exhibit distinctive characteristics, including extensive self-renewal and the ability to differentiate generating phenotypically diverse progeny. Breast cancer stem cells (BCSCs) drive tumor progression, metastatic dissemination, drug resistance, and cancer relapse (Lim et al., 2021).

CSCs exhibit a diverse array of functional and biological properties that distinguish them from non-cancer stem cells in tumors. CSCs are mainly characterized by the presence or absence of diverse stem cell markers whose levels are heterogeneously distributed among tumors. These markers range from cell surface protein, cytoplasmic enzymes, and nuclear transcription factors (Lim et al., 2021).

**FIGURE 1:**
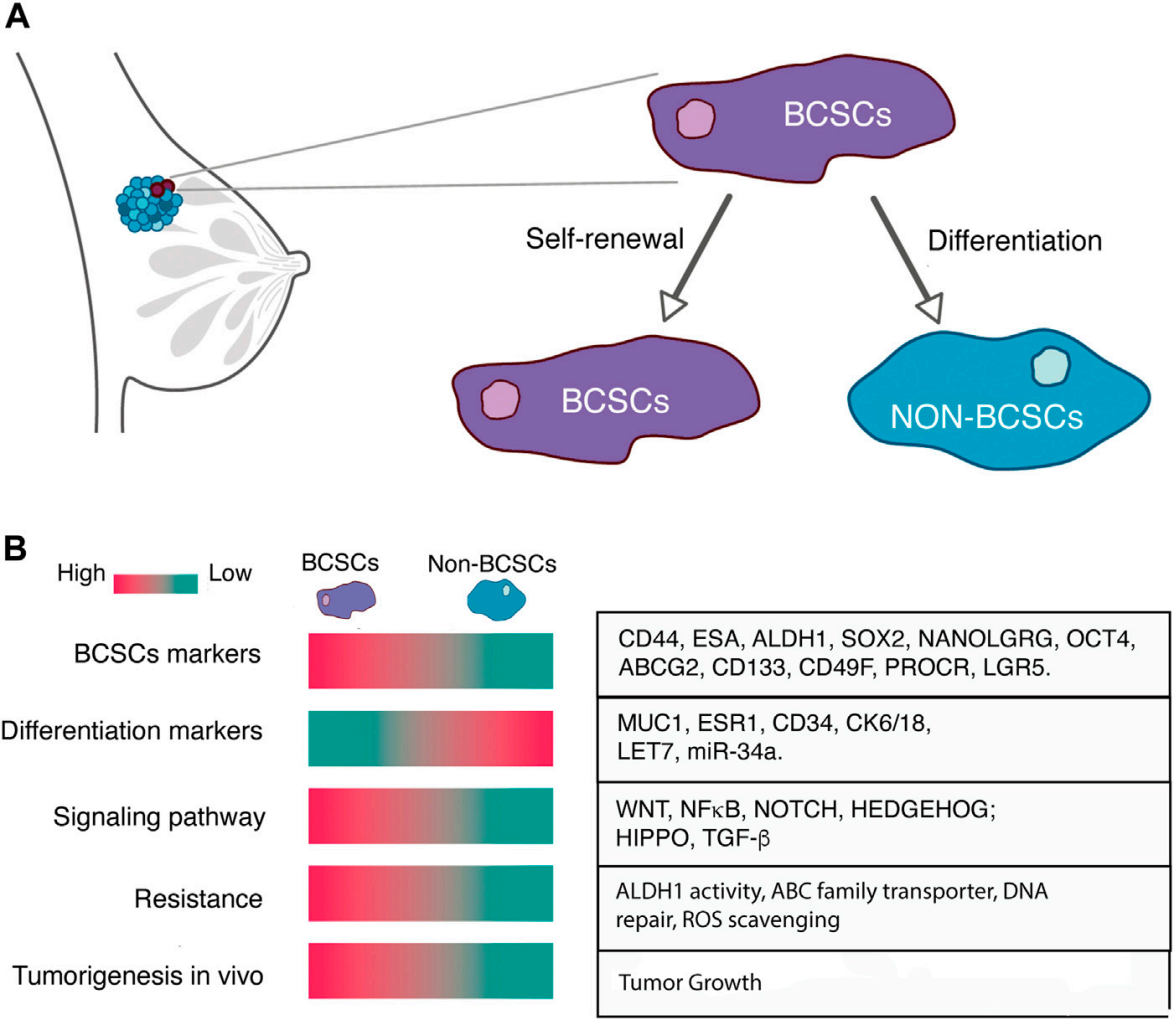
**(A)** Breast cancer stem cells represent a distinct subset of tumor cells characterized by their remarkable capacity for self-renewal and their ability to differentiate into non-BCSCs. **(B)** BCSCs can be distinguished by the high expression of BCSCs markers, low expression of differentiation markers, specific signaling pathways activity, resistance to conventional therapy and the high tumorigenesis potential *in vivo*.

BCSCs are characterized by the high expression of CD44, ESA, and low or absent levels of CD24. In addition to CD44+/ESA+/CD24-cells, other studies have identified and isolated BCSCs using CD133, CD49f, PROCR, LGR5, ABCG2, among others. BCSCs also exhibit high levels of transcription factors such as SOX2, NANOG, and OCT4, which are crucial for maintaining stemness and preventing differentiation. Moreover, BCSCs present high activity of the aldehyde dehydrogenase 1 cytosolic enzyme (ALDH1) and high expression of ABC family transporters, which provide resistance to conventional cancer treatments (Wang et al., 2022) (Figure 1).

Similarly, another crucial attribute that sustains breast cancer stem cells is their metabolic flexibility and adaptation. Most BCSCs display a heightened glycolytic rate, exhibit increased glucose uptake, and lactate production, and a decreased mitochondrial respiration. Recent evidence has indicated that BCSCs possess the ability to alternate between glycolysis and mitochondrial oxidative phosphorylation (OXPHOS) in the presence of oxygen, enabling them to facilitate tumor growth. This metabolic flexibility enables CSCs to engage in mitochondrial respiration and generate ATP, thereby conferring a survival advantage under conditions where glycolysis is compromised. Interestingly, proliferative BCSCs prefer the OXPHOS metabolism, while quiescent BCSCs opt for a glycolytic metabolism. In addition, CSCs also rely on mitochondrial fatty acid oxidation as an alternative energy source to maintain their survival, self-renewal, and chemoresistance properties. CSCs exhibit dysregulated fatty acid synthesis, which leads to increased lipid production and accumulation, thereby supporting the biosynthesis of macromolecules vital for cellular growth and division. Moreover, CSCs demonstrate distinctive alterations in glutamine metabolism, utilizing it as a carbon source to fuel energy production and sustain the biosynthesis of essential macromolecules. These distinctive metabolic features of CSCs enable them to adapt to the challenging conditions within the tumor microenvironment, promote self-renewal, and drive tumor progression (Gao and Dong, 2020).

Functionally, CSCs can grow tumors retaining the self-renewal ability in several serial passages even when they are transplanted in very low numbers. In addition, CSCs have the ability to grow in low adherence conditions and exhibit improved processes of invasion and metastasis (Zhang et al., 2022). The characterization of CSCs has been a key step in cancer research, huge efforts are undertaken to decipher the complex biology of CSCs. Tremendous efforts are being undertaken to further elucidate the unique features of CSCs. Novel therapeutic strategies are being proposed to eliminate the CSC fraction, overcome drug resistance, and prevent cancer relapse. Currently, one of the promising approaches to prevent CSC maintenance is based on blocking the various signaling pathways that maintain stemness in breast cancer.

This review aims to provide an overview of the main signaling pathways involved in the maintenance of BCSCs. We also summarized the current therapeutic advances to target BCSCs.

## 2 Signaling pathways regulating breast cancer stem cells

**FIGURE 2:**
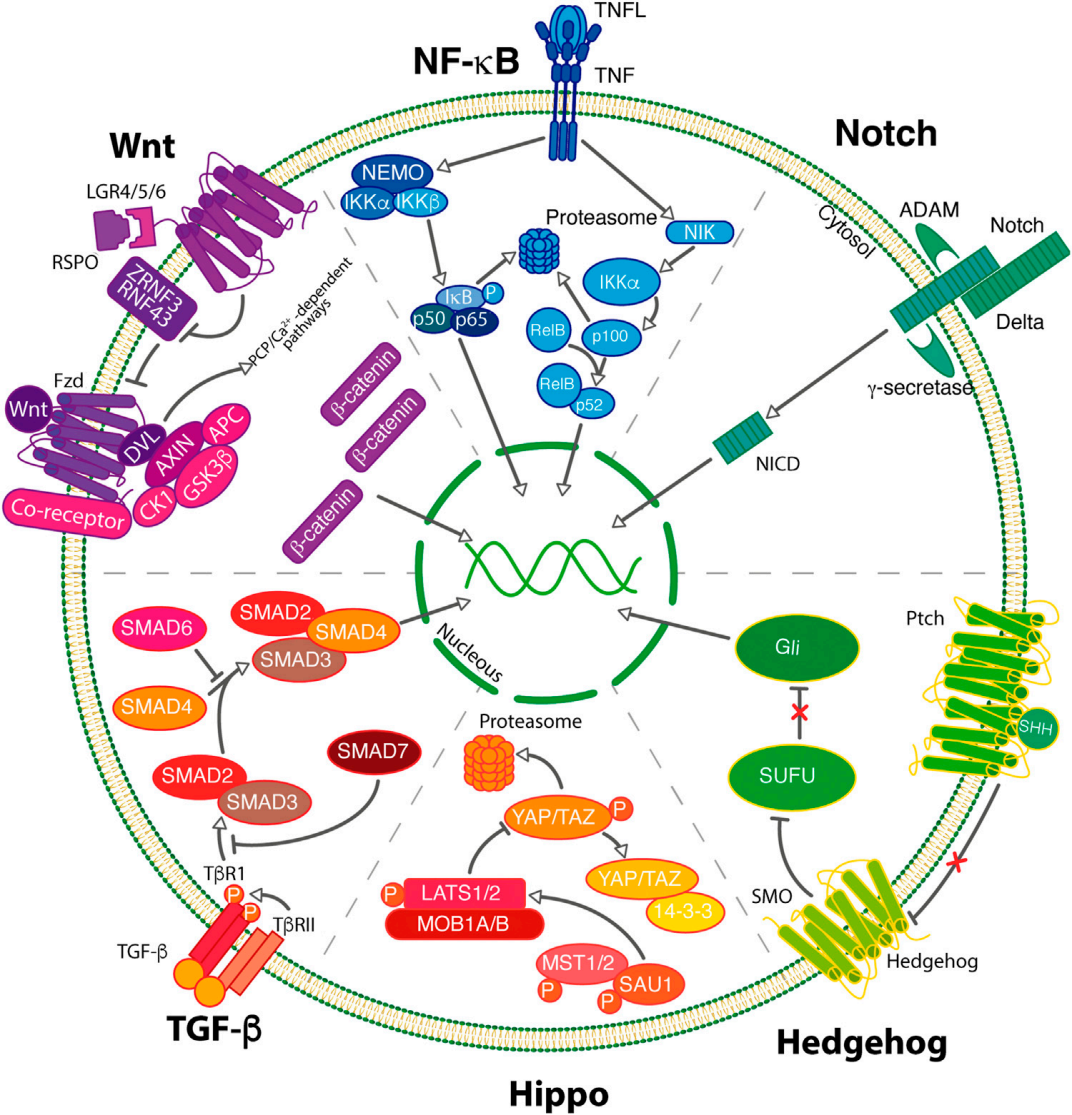
BCSCs maintenance can be regulated by the activity of signaling pathways. In breast cancer, pivotal signaling pathways including Wnt, NF-κB, Notch Hedgehog, Hippo and TGF-β govern the self-renewal process of BCSCs.

The properties of CSCs are modulated by intricate signaling pathways, which modulate the stemness, self-renewal, differentiation, proliferation, and survival of CSCs. The main signaling pathways involved in CSC maintenance include Notch, Wnt, Hedgehog, NF-KB, Hippo among others. These pathways play important roles in the invasion, metastasis, autophagy, and EMT. The signaling molecules and components of these pathways have been extensively studied as therapeutic target options to destroy CSCs (Figure 2).

### 2.1 WNT signaling pathway

The WNT signaling pathway is a highly complex and evolutive conserved pathway involved in the regulation of cell fate determination, cell polarity, and self-renewal of normal and CSCs. The Wnt family comprises 19 secreted cys-rich glycoproteins, which can activate more than 15 cell membrane receptors. This pathway is triggered through Wnt ligands, Frizzled receptors, and/or low-density lipoprotein-related protein (LRP) 5 and LRP6 coreceptors. WNT signaling typically employs two major pathways: canonical (mediated through β-catenin) and non-canonical pathways (independent of β-catenin) (Xu et al., 2020).

In the context of canonical Wnt signaling, non-active conditions enable the continuous phosphorylation of β-catenin mediated by the inhibitory complex of β-catenin, which includes AXIN, GSK3β, APC, and CK1. This phosphorylation triggers the proteasomal degradation of β-catenin through the E3 ubiquitin ligase β-TrCP. In contrast, under active conditions, Wnt ligands (Wnt1, Wnt2, Wnt3, Wnt3a, Wnt8b, Wnt10a, Wnt10b, among others) bind to their receptors and induce the phosphorylation of the co-receptor LRP5/6. This phosphorylation event recruits both the Dishelved protein (Dvl) and Axin, thus preventing the inhibition of β-catenin phosphorylation. Subsequently, the accumulation of β-catenin levels results in its nuclear translocation, where it binds to TCF/LEF transcription factors to activate the transcription of numerous Wnt target genes (Xu et al., 2020).

It has been reported that molecules such as LGR4/5/6 receptors enhance the Wnt/β-catenin signal by interacting with R-spondin ligands (RSPOs). In the absence of RSPOs, the E3 ubiquitin ligases ZNRF3 and RNF43 continuously ubiquitinate the Wnt receptor complex, thus inducing their degradation. The activation of LGR4/5/6 by RSPOs ligands neutralizes the ZNRF3 and RNF43 ligases allowing for the stabilization of surface Frizzled receptors and thus boosting the WNT signaling (Ter Steege and Bakker, 2021).

Non-canonical Wnt signaling is independent of β-catenin. This pathway is activated when Wnt ligands bind to Frizzled and other co-receptors such as ROR1, ROR2, or RYK. The non-canonical signaling induces the activation of PCP, RTK, or Ca++ signaling cascades. This pathway induces several downstream effectors, including multiple small Rho GTPases, Jnk, Src, NLK and heterotrimeric G proteins. Activation of this pathway results in transcriptional regulation and cytoskeletal rearrangement (Xu et al., 2020).

In breast cancer, both canonical and non-canonical WNT signaling participates in the regulation of CSCs. Breast tumors exhibit constitutive activation of the Wnt/β-catenin pathway, accompanied by increased nuclear stabilization of β-catenin. Even though Wnt signaling molecules harbor some mutations, several studies have identified that most alterations frequently occur in activators or inhibitors of the Wnt signaling pathway. Activators of the Wnt/β-catenin pathway such as Wnt and Dvl ligands are commonly amplified or overexpressed, while Wnt inhibitors such as FRP1, DKK1, and APC are commonly inactivated. (Lindvall et al., 2007; Feng et al., 2018). Sustained Wnt-β-catenin activation endows CSCs with self-renewal abilities, proliferation, invasion, and metastatic abilities (Katoh, 2017).

Several studies have highlighted the importance of the Wnt/β-catenin signal in the maintenance of different BCSCs subpopulations. The canonical pathway has been associated with self-renewal, mammosphere formation, migration, invasion, and drug resistance. Li et al., showed that BCSCs exhibiting elevated expression of the transcription factor Twist, are regulated through the Wnt/β-catenin signal (Li and Zhou, 2011). In addition, activation of Wnt/β-catenin triggered by histone deacetylase inhibitors, provokes an increment in the population of ALDH-positive BCSCs, resulting in an enhanced ability to grow spheres and to seed tumors (Debeb et al., 2012). Some other studies have found that different subsets of BCSCs depend on the Wnt/β-catenin signaling to maintain their properties and self-renewal potential (Zhao Z. et al., 2014; Siddharth et al., 2017; Zhu L. et al., 2019).

LGR4/5/6 receptors and their RSPO ligands induce stemness in breast cancer. These receptors have been involved in the maintenance of the stem cell phenotype through the potentiation of Wnt/β-catenin signaling. It has been shown that the LGR4 receptor promotes tumorigenesis, induces the epithelial-mesenchymal transition process, and favors a stem cell phenotype through the Wnt/β-catenin pathway (Yue et al., 2018). LGR5 also potentiates the Wnt/β-catenin signal resulting in the acquisition of a stem cell phenotype. Notably, LGR5 has been associated with a worse prognosis in patients with breast cancer (Yang et al., 2015; Hou et al., 2018; Chen and Xue, 2019). Similarly, LGR6 promotes the proliferation and metastasis of breast cancer cells through the Wnt/β-catenin signal. LGR6 is commonly overexpressed in triple-negative breast cancer tumors and has been associated with a worse prognosis in patients with this disease (Kong et al., 2020).

Besides the classic molecules participating in the Wnt/β-catenin signaling, various non-classical activators have been associated with the maintenance of stemness in breast cancer. Pigo2 protein (Pygopus Family PHD Finger 2) acts as a scaffold for the recruitment of β-catenin, allowing it to associate with histone methyl and acetyltransferases, favoring the transcription of genes associated with stemness, and thus increasing the BCSC fraction (Chen et al., 2010). Another study indicates that the cell cycle regulator p21 is capable of regulating the stemness of breast cancer cells through the Wnt/β-catenin signaling. p21 induces the expression of Cyclin D1 and TCF1, a transcription factor involved in the Wnt pathway, leading to the activation of the Wnt/β-catenin pathway and the induction of BCSCs self-renewal (Benard et al., 2019). Tang et al., showed that the enzyme β1, 4-Galactosyltransferase B (B4galT5) regulates the stemness phenotype of breast cancer. B4galT5 protects the Frizzled-1 receptor from degradation via the lysosome, allowing its membrane stabilization and favoring the Wnt/β-catenin pathway activation (Tang et al., 2020). The BOP1 molecule regulates drug resistance and stemness phenotype in triple-negative breast cancer cells. BOP1 allows the recruitment of the CBD protein, facilitating β-catenin acetylation, thus inducing its activation, and increasing the expression of stem cell markers such as CD133 and ALDH1A1 (Li et al., 2021). Similarly, salt-inducible kinase 2 (SIK2) is capable of regulating the stem cell phenotype of breast cancer by favoring the phosphorylation of the CK1 protein kinase and the co-receptor of the Wnt/LRP6 signaling, facilitating the Wnt/β-catenin activation (Rong et al., 2022).

Similarly, various non-coding RNAs have been associated with the maintenance of stemness in breast cancer through the Wnt/β-catenin signal. It has been well established that Wnt/β-catenin induces the expression of the lncRNA Lin28 to block let7 miRNA activity, thus promoting a stem cell phenotype in breast cancer cells (Cai et al., 2013). In addition, the microRNA mir-204 can activate the Wnt/β-catenin signal through the Sam68 protein, thus regulating stem cell self-renewal and tumorigenesis in SKBR3 and MCF7 breast cancer cell lines. (Wang et al., 2015). Eterno et al., showed that the protein AurkA can regulate the Wnt3a ligand levels by inhibiting mir-128 and thus regulating the stem cell phenotype in breast cancer (Eterno et al., 2016). It was discovered that the lncRNA LncCCAT1 promotes stemness in breast cancer by interacting with the miR-204/211, miR-148a/152, and Annexin A2, inducing the overexpression of TCF4 and favoring the activation of the Wnt/β-catenin signal (Tang et al., 2019). LUCAT1 is associated with advanced breast cancer tumors and is expressed mainly in the stem fraction, where it regulates the self-renewal of BCSCs by acting as a mir-5582-3p sponge, favoring the expression of TCF7L2 and promoting the Wnt/β-catenin signal. (Zheng et al., 2019). In addition, the mir-5188 inhibits the expression of FOXO1 which facilitates the ubiquitination of β-catenin. Blockage of FOXO1 by mir-5188 results in the accumulation of β-catenin and induces the activation of Wnt signaling thus promoting the self-renewal and maintenance of CSCs (Zou et al., 2019). The lncRNA THOR is also able to regulate stemness by interacting and stabilizing the β-catenin mRNA and increasing its expression, favoring the activation of the Wnt signaling (Wang et al., 2020). Taken together, these findings indicate that lncRNAs, miRNAs, and other molecules can influence canonical WNT/β-catenin signaling and this is involved in the maintenance and expansion of BCSCs.

Although less studied than the WNT/β-catenin pathway, non-canonical signaling also plays a role in the regulation of the BCSC fraction. In CSCs, the non-canonical pathway is commonly activated by Wnt5a, and other non-canonical ligands secreted from cancer or stromal cells. Wnt5a enhances the sphere formation efficiency by activating the non-canonical Wnt pathway in MMTV-Wnt1 mouse primary cells. The effects of Wnt5a on stemness depend on the receptor tyrosine kinase (ROR2) that binds Wnt5a and transduces the Wnt signal, thus inducing the activation of the Jun N-terminal kinase (JNK) (Many and Brown, 2014). Interestingly, breast cancer patients expressing ROR2 had shorter overall survival than those harboring tumors without ROR2 expression (Henry et al., 2015). Although Wnt5a can activate the Wnt/β-catenin in special circumstances (Mikels and Nusse, 2006; van Amerongen et al., 2008), some studies have shown that the effects of Wnt5a on stemness depend on the non-canonical Wnt and not on the canonical signaling (Many and Brown, 2014). Notably, Wnt5a is overexpressed specifically in basal breast cancer cell lines (MDA-MB-231 and BCap-37), which harbor a mesenchymal phenotype and a high proportion of CSCs. Remarkably, the inhibition of Wnt5a mediated by the disruption of Twist-BRD4 association suppresses CSC properties, reduces the invasion, and impairs tumorigenesis of basal breast cancer cells (Li and Zhou, 2011). Wnt5B has also been involved in the regulation of stemness via the interaction of the Fzd7 receptor and the subsequent activation of the non-canonical Wnt pathway. Fzd7 knockdown reduces the fraction of LGR5+ CSCs, suppresses tumorigenesis, and impairs metastasis. Mechanistically, Fzd7/Wnt5b regulates the expression of key intracellular molecules such as phosphorylated Stat3, Smad3, and Yap1 to induce EMT and stemness. Interestingly, Col6a1 is implicated in the Fzd7-Wnt5b signal and mediates the stemness effect of Fzd7/Wnt5b (Yin et al., 2020). Wnt5a and Wnt5b can also interact with Fzd2, a receptor that signals to the non-canonical via. Interestingly, Fzd2 and its ligands are overexpressed in high-grade tumors, and metastatic cancer cell lines, and high Fzf2 expression is associated with shortened overall survival, relapse-free survival, and distant metastasis-free survival (Gujral et al., 2014; Yin et al., 2020). Fzd2 also correlates with the expression of EMT markers and promotes EMT, drug resistance, and induces stemness via non-canonical Wnt signaling. Interestingly, Fzd2 knockdown impairs stemness, reduces the fraction of Lgr5+ CSC subpopulation, inhibits migration and invasion, impairs tumor growth, and enhances drug sensitivity (Gujral et al., 2014; Yin et al., 2020). Interestingly, the effect of Fzd2 in the induction of EMT and cell migration is mediated by the association with Stat3. Mechanistically, Fzd2 is phosphorylated on Tyr552 resulting in the binding of Fzd2 to the SH2 domain of Fyn kinase, which activates Star3 via the phosphorylation of Tyr705 (Gujral et al., 2014). Interestingly, Fzd2/Wnt5a/b regulate stemness by activating several oncogenic pathways including IL6/STAT3, Yap1 and TGF-b1/Smad3 (Yin et al., 2020).

### 2.2 NF-κB signaling pathway

NF-κB is a family of transcription factors that regulates the expression of genes associated with immune response, inflammation, survival, cell differentiation, and stemness. The family consists of five members: RelA (p65), RelB, c-Rel, NFκB1 (p105/p50), and NFκB2 (p100/p52). These proteins harbor a conserved N-terminal Rel homology domain (RHD) which facilitates homo or heterodimerization, nuclear localization, and DNA binding. In addition, only RelA, Relb, and c-Rel contain a C-terminal transactivation domain (TAD) which mediates coactivators interactions to promote gene transcription. Remarkably, p50 and p52 lack TAD domain, thus p50/p50, p50/p52 or p52/p52 dimers fail to activate gene expression. The precursor of p50 (p100) and p52 (p105) proteins contains ankyrin repeats which are proteolytically cleaved to generate the active p50 and p52 proteins (Vazquez-Santillan et al., 2015). Members of the NF-κB family possess the ability to generate distinct homo or heterodimers. The formation of NF-κB dimers varies depending on the specific cellular context, and the abundance of these molecules. For instance, the p50/RealA and p52/RelB dimers are known to be the major players in the canonical and non-canonical pathways, respectively. Similarly, the activation of the cRel/p50 heterodimer is a crucial element of the innate immune response and the subsequent eradication of pathogens (Courtine et al., 2012).

The NF-κB members are retained in the cytoplasm by a family of NF-κB inhibitor (IκB) proteins (IκBα, IκBβ, and IκBε) and other proteins harboring ankyrin repeats. Upon receiving an activation stimulus, NF-κB proteins form homo or heterodimers, which translocate to the nucleus and regulate the expression of their target genes (Vazquez-Santillan et al., 2015). NFκB is activated by two major signaling branches: the canonical and the non-canonical pathway.

The canonical signaling is rapidly inducible and independent of protein synthesis, this pathway is associated with immunological and inflammatory roles. This signal is activated by diverse external stimuli promoting inflammation, such as proinflammatory cytokines including tumor necrosis factor α (TNF-α), pathogen-associated molecular patterns (PAMPs), and damage-associated molecular patterns (DAMPs). The interaction of ligands with their receptors favors the recruitment of proteins leading to the activation of the inhibitory kappa B kinases (IKK) complex, consisting of the scaffold protein NF-κB essential modulator (NEMO) and two catalytic subunits, IKKα and IKKβ. Upon activation, the IKK complex catalyzes the phosphorylation of the NF-κB inhibitor (IκB) proteins, triggering IκB polyubiquitination and subsequent degradation in the proteasome. Under non-active conditions, IκB proteins bind to NF-κB dimers, sequestering NF-κB and preventing their nuclear translocation, thus IκB phosphorylation mediated by IKKs is essential to NF-κB nuclear translocation and to modulate the expression of target genes (Vazquez-Santillan et al., 2015).

In contrast to canonical signaling, the non-canonical pathway is slow, persistent, and depends on the novo protein synthesis. Non-canonical signaling is associated with the differentiation, development, and survival of immune cells. This pathway is activated by a number of ligands including CD40 ligand (CD40L), B cell activating factor (BAFF), receptor activator of nuclear factor kappa B ligand (RANKL), and lymphotoxin β (LTβ). These ligands interact and bind to a subset of receptors favoring the stabilization of the NF-B-inducing kinase (NIK), which in turn phosphorylates the IKK complex formed exclusively by IKKa homodimers. The IKK complex phosphorylates the p100 NF-κB protein provoking its partial degradation via the proteasome, which converts p100 to the active p52 form. P52 forms dimers with RelB or p65, which can translocate to the nucleic fraction and favors the expression of its target genes (Vazquez-Santillan et al., 2015).

In BCSCs, constitutive activation of the NF-κB pathway has been observed in MCF7 and MDA-MB-231 breast cancer cell lines. Both canonical and no canonical pathway participates in the chemoresistance, tumorigenesis, and self-renewal of BCSCs (Pratt et al., 2009; Liu et al., 2010; Kendellen et al., 2014; Jia et al., 2015; Kumar et al., 2021). Interestingly, accumulating evidence has shown that NF-κB activity is able to expand the BCSCs (Yamamoto et al., 2013; Kendellen et al., 2014) and regulate the expression of stem cell markers (Smith and Cai, 2012). It has been shown that canonical and non-canonical NF-κB signaling is required by CSCs to self-renew and to form tumors in murine *in vivo* models. Interestingly, NF-κB regulates stemness by promoting the epithelial to mesenchymal transition and the expression of inflammatory cytokines Interleukin 1B and interleukin 6 (Kendellen et al., 2014).

Canonical NF-κB signaling drives resistance to chemotherapy in quiescent BCSCs and its pharmacological blocking sensitizes breast tumors to chemotherapy (Kumar et al., 2021). The inhibition of IKKβ or NF-κB subunits disrupts mammosphere formation and impaired stemness of SUM149 (Kendellen et al., 2014). Interestingly, the super repressor mutant of the IKB protein inhibits the stem cell properties, reduces proliferation, and suppresses the clonogenicity and tumorigenic ability of breast tumor cells by constitutively inhibiting the canonical NF-κB pathway (Liu et al., 2010; Hinohara et al., 2012). IKKα, a kinase involved in both canonical and non-canonical signaling, also contributes to the maintenance of the BCSCs fraction. The employment of an inactive mutation of IKKα impairs self-renewal and delays tumor formation in MMTV-c-neu mice (Cao et al., 2007).

It has been well demonstrated that non-canonical NF-κB also exerts essential roles in the maintenance and self-renewal of BCSCs. The non-canonical NIK protein is preferentially expressed in BCSCs, increases the expression of stem cell markers, regulates the self-renewal and expansion, and promotes tumorigenesis of CSCs through IKKα (Zhang et al., 2013; Vazquez-Santillan et al., 2016). In the same way, the IKKε kinase is able to promote the stem cell phenotype of breast cancer cell lines including MCF7 (Orlova et al., 2019).

Furthermore, NF-κB ligands such as TNF-α and RANKL increase the proportion of BCSCs by promoting the activity of the NF-κB pathway. TNF-α induce the expression of TAZ through the non-canonical NF-κB pathway and increases the proportion of BCSCs. Mechanistically p52 binds to the promoter region of TAZ to favor its transcription. TNF-α/TAZ plays a crucial role in the maintenance of BCSCs (Liu W. et al., 2020). The receptor activator of nuclear factor kappa B ligand (RANKL) and its receptor (RANK) participate in the activation of the non-canonical NF-κB pathway. RANK expression levels have been associated with poor prognosis in breast cancer patients. Accumulating evidence has shown that the RANK receptor increases tumorigenesis, migration, epithelial-mesenchymal transition, resistance to therapy, and stemness in breast cancer (Palafox et al., 2012; Pfitzner et al., 2014; Renema et al., 2016; Cuyàs et al., 2017). Interestingly, the inhibition of RANKL by the RANK-Fc recombinant protein results in the reduction of breast cancer tumorigenesis and the induction of the differentiation of CSCs, suggesting that the RANKL/RANK signaling expand the CSC fraction by activating the NF-κB signaling (Yoldi et al., 2016).

NF-κB signaling also renders BCSCs with invasive and metastatic abilities. A study found that Lin28, a downstream effector of IKKβ, enhances the metastatic abilities of BCSCs. IKKβ inhibition reduced the expression of stem cell factors (LIN28, OCT4, SOX2, and NANOG) and eliminate the ability of CSCs to metastasize (Chen et al., 2015). EMT induction mediated by NF-κB signaling also contributes to the invasive, tumorigenic, and metastatic abilities of BCSCs. Inhibition of NF-κB reverts the EMT and decreases invasion, reduces metastasis, and restores cell sensitivity to chemotherapy (Asiedu et al., 2014; Kendellen et al., 2014).

A plethora of molecules activating the NF-κB signaling has been shown to regulate the BCSC fraction. Recent evidence found that the receptor GPR50 is highly expressed in BCSCs and regulates the activity of the NF-kB pathway, enabling CSCs to form spheres, proliferate and migrate. The heat shock protein Hsp27 participates in the regulation of the epithelial-mesenchymal transition process and promotes stemness through the NF-κB signal (Wei et al., 2011). Neuropilin 1 (NRP1) is expressed in BCSCs and induces stemness by stimulating the NF-κB signaling (Glinka et al., 2012). Another study showed that the transcription factor FOXA1 inhibits stemness by blocking the expression of Interleukin 6 through the inhibition of NF-κB recruitment to the IL6 promoter gen (Yamaguchi et al., 2017). Stromal cell-derived factor 1 (SFD-1) promotes the stem cell phenotype, cell proliferation, migration, and invasion through the NF-κB signal (Kong et al., 2016). Another study found that the let7 miRNA inhibits the ability of CSCs to form mammospheres and impairs tumorigenicity by disrupting the NF-κB and MAPK signaling, suggesting that let7 regulates the stem properties in breast cancer (Xu et al., 2015). The microRNAs 221/222 promote the stem cell phenotype through the inhibition of PTEN and the activation of the AKT/NF-κB/COX-2 pathway (Li et al., 2017).

The NF-κB pathway was initially characterized as an inductor of inflammation and a regulator of the immune system in normal processes and cancer. However, it has been recently shown as an essential signal for developmental processes and as an important promoter of stemness in breast cancer, making it an attractive target to deplete BCSCs and improve the prognosis of breast cancer patients.

### 2.3 Notch signaling pathway

Notch is an evolutionarily conserved signaling pathway exerting pivotal roles in proliferation, cell fate determination, differentiation, and stem cell maintenance. In mammals, this pathway consists of 5 notch ligands (Jagged1, Jagged2, Delta-like (DLL) 1, 3, and 4) and 4 notch receptors (Notch 1-4). The notch pathway is activated when the extracellular domain of the Notch receptor binds to Notch ligands. Upon ligand binding, Notch receptors undergo two proteolytic cleavages, the first cleavage is performed in the extracellular region and catalyzed by the ADAM family of metalloproteases, while the second occurs in the intracellular region mediated by the y-secretase enzyme complex (presenilin, nicastrin, PEN2, and APH1). These cleavages provoke the release of the Notch intracellular domain (NICD) from the membrane and the subsequent translocation into the nucleus, where it forms a complex with CSL and a member of the Mastermind (MAM) family of coactivators to regulate the transcription of Notch target genes (Miele et al., 2006; Harrison et al., 2010a).

It is well known that the notch signal is an important regulator of normal mammary stem cells (Farnie and Clarke, 2007). Aberrant expression of Notch receptors has been observed in breast cancer and is associated with poor prognosis. Notch 1 and Notch 4 are enriched in BCSCs compared to differentiated cells, both receptors have been reported to regulate breast cancer stem cells. Remarkably, Notch 4 exerts a stronger effect on the maintenance of BCSCs (CD44^+^/CD24^-^/ESA^+^). Notch 1 and 4 inhibition impair breast cancer stem cell activity by reducing ALDH activity thus reducing tumor growth and render CSCs resistant to drug therapy. This has been observed in MCF7, T47D and ZR75-1 breast cancer cell lines (Harrison et al., 2010b; Simões et al., 2015).

Notch signaling increases cell proliferation, angiogenesis, apoptosis, and stimulates drug resistance, EMT, metastasis, and increases BCSC numbers. Hyperactivation of notch signal activity has been found in BCSCs, this pathway regulates the properties of a large number of BCSCs. (Wu, 2007; Wong et al., 2012; Lagadec et al., 2013; Peng et al., 2014; D’Angelo et al., 2015; Acar et al., 2016; Pal et al., 2017; Kontomanolis et al., 2018).

A study reported that Notch signaling induces stemness by promoting the deacetylation and subsequent activation of ALDH1A1 (Zhao D. et al., 2014). Additionally, the Notch signal highly correlates with Ki-67 expression in BCSCs (Cui et al., 2015). Notch signal induced by radiation or hypoxia leads to an acquisition of a breast cancer stem cell phenotype (Xing et al., 2011; Wong et al., 2012; Lagadec et al., 2013).

Different molecules regulate stemness in breast cancer through the Notch signal. Majumder et al., demonstrated that Cox-2 is capable of inducing BCSCs with high ALDH activity by promoting Notch expression and activating Notch signaling in MCF7 and SKBR3 cell lines (Majumder et al., 2016). Another study revealed that Mel-18 blocks the Notch signal by inhibiting the expression of the Jagged-1 ligand and thus reducing BCSCs (CD44^high^/CD24l^ow^) in MCF7 cells (Won et al., 2012). Likewise, Garcia-Heredia et al., showed that Numbl inhibition induces Notch activity and promotes the acquisition of a stem cell phenotype in the T47D cell line (García-Heredia et al., 2016). Additionally, MAPK17 kinase interacts with NUMB, a notch inhibitor, and facilitates the activation of the Notch pathway which in turn expands the BCSC fraction (Garcia-Heredia et al., 2017). The SATB1 molecule is also able of activating the Notch signal and regulating the stem phenotype in breast cancer (Sun et al., 2015). Some microRNAs such as mir-129 decrease the Notch signal by suppressing the expression of Cyclin d1/DICER and thus inhibiting the stem cell phenotype. Mir526b-3p is commonly reduced in breast cancer, when expressed, it regulates the Hif2a/Notch signal and inhibits stemness (Yan et al., 2018). Mir34a also downregulates the Notch1 receptor, inhibits stemness, and renders cancer cells more sensitive to paclitaxel (Kang et al., 2015).

In line with previous observations, Notch signaling induces the expansion of the BCSCs, suggesting that inhibitors of this signaling system could decrease this cell population and improve therapy response in these tumors.

### 2.4 Hedgehog signaling pathway

The Hedgehog pathway (HH) is a signaling system involved in tissue homeostasis, embryogenesis, development, and regeneration. Hedgehog molecules constitute a small family of secreted signaling proteins including Sonic Hedgehog (SHH), Indian Hedgehog (IHH), and Desert Hedgehog (DHH). The Patched receptor (PTCH), the transmembrane protein Smoothened (SMO), and Gli transcription factors (Gli 1, 2 and 3) along with HH ligands are the major players in Hedgehog signaling (Bhateja et al., 2019). The Gli code has been proposed as a phenomenon where the collaborative action of three transcription factors, namely Gli1, Gli2, and Gli3 with their respective activating and repressing functions, is necessary for the integration of the Hedgehog signaling pathway within the cells. The Gli code undergoes modifications when HH ligands are present, resulting in the transcription and activation of Gli1 and the inhibition of Gli2 and Gli3 processing (Ruiz i Altaba et al., 2007). Mechanistically, in the absence of HH ligands, the patched receptor inhibits the smoothened receptor, and gene expression is repressed by Gli1 and Gli2. Upon ligand binding to PTCH, the repression of SMO is relieved, allowing the activation of GLI proteins to facilitate the transcription of target genes (Bhateja et al., 2019).

The Hedgehog pathway is largely inactive in most postnatal tissues, but this is commonly activated in cancer. Recent finding has demonstrated that the Gli code not only regulates stemness but also plays a crucial role in tumor progression and the development of metastatic lesions (Ruiz i Altaba et al., 2007). This pathway favors tumor progression and is associated with aggressive tumors with high CSC content in breast cancer (Kasper et al., 2009; Zhao et al., 2016; Riobo-Del Galdo et al., 2019). Accumulating evidence indicates that Hedgehog signaling plays a role in the regulation of CSC properties by promoting self-renewal, stemness, and drug resistance in breast tumors (Liu et al., 2006; Tanaka et al., 2009; He et al., 2015; Sims-Mourtada et al., 2015).

Different studies revealed that PTCH1, GLI1, GLI2, and SMO are highly expressed in the CSC fraction and their expression reduces upon stem cell differentiation. Activation of Hedgehog signaling promotes tumorigenesis and metastasis, increases self-renewal, proliferation, and sphere forming-efficiency of BCSCs via SHH-mediated upregulation of the polycomb protein Bmi-1 (Liu et al., 2006; Wang L. et al., 2014).

Notably, CD24, a protein absent or low expressed in BCSCs, decreases the stem cell phenotype by inhibiting the expression of SHH and GLI1, and deactivating the Hedgehog pathway (Suyama et al., 2016). In addition, various molecules regulate stemness by potentiating the Hedgehog signal. Yuan Cao and collaborators showed that glutamic-pyruvic transaminase (GPT2) increases stemness by reducing α-ketoglutarate levels and inhibiting the enzyme proline hydroxylase 2 (PHD2) involved in the regulation of HIF1a stability. Accumulation of HIF1α levels results in the constitutive activation of the SHH signaling (Cao et al., 2017). Similarly, p63 regulates the expression levels of SHH, GLI2, and PTCHD1, thus facilitating their activity and expanding the number of BCSCs (Memmi et al., 2015). Otherwise, the transcription factor FOXC1 (Forkhead box C1 protein) mediates the activation of the SMO-independent Hedgehog signal by interacting and activating Gli2, thus inducing the activity of ALDH1 and promoting the self-renewal of CSCs in basal breast cancers (Han et al., 2015, 2016). Moreover, the ETV4 transcription factor also activates the Hedgehog signaling by promoting the expression of CXCR4 and thus enriching stemness by favoring the glycolytic activity of BCSCs (Zhu et al., 2021). The enzyme 24-dehydrocholesterol reductase (DHCR24) also expands the BCSC fraction through the activation of the HH signaling (Qiu et al., 2020). Another study found that tetraspanin 8 (TSPAN8) interacts with PTCH1, stabilizing its membrane location, and subsequently favoring BCSCs, drug resistance, and tumorigenesis by inducting the activity of hedgehog signaling (Zhu R. et al., 2019). Circ_DCAF6 RNA is also able to promote HH signaling by inducing GLI1 expression through sequestering mir-616-3p and thus expanding the CSC fraction (Ye et al., 2020).

Collectively, these data suggest that Hedgehog signaling induces stemness, regulates self-renewal, and participates in the CSC-driven propagation of breast cancer. Since HH signaling exerts profound implications in the expansion of CSCs, molecules disrupting this pathway are ideal therapeutic targets to reduce the fraction of BCSCs to achieve a durable clinical response.

### 2.5 Hippo signaling

Hippo signaling is an evolutive conserved pathway that regulates development, tissue homeostasis, and organ size. The hippo pathway consists of both a kinase cascade (MST and LATS) and a downstream transcriptional module (YAP and TAZ). The kinases are composed of MST1 and MST2, which phosphorylate and activate downstream kinases LATS1 and LATS2, and their scaffold MOB1A/B. The hippo signaling pathway is activated when MST1/2, LATS1/2, and MOB1A/B are phosphorylated. Hippo activation results in the inactivation of the transcriptional coactivators YAP and TAZ mediated by LAST1/2 phosphorylation. Phosphorylated of YAP and TAZ results in their localization in the cytoplasm through binding to 14-3-3 protein, followed by their degradation in a ubiquitin-proteasome-dependent manner. (Maugeri-Saccà and De Maria, 2016; Wu and Guan, 2021). Conversely, when the hippo pathway is inactivated, dephosphorylated YAP and TAZ translocate to the nucleus and through the TEAD family of transcription factors, induce gene expression.

Hippo signaling has been associated with normal mammary development. In cancer, YAP/TAZ act as oncogenes promoting proliferation, invasion, migration, epithelial-mesenchymal transition, metastasis, and BCSCs self-renewal (Shi et al., 2014; Maugeri-Saccà and De Maria, 2016). Accumulated evidence has shown that YAP/TAZ signaling regulates BCSC maintenance. TAZ is overexpressed in breast cancer and indispensable to promoting the self-renewal of BCSCs (Cordenonsi et al., 2011). Bartucci et al., (2015) shows that TAZ is an important mediator of metastasis, chemo-resistance and tumorigenesis of BCSCs. Chang et al., 2015 show that TAZ regulated BCSCs self-renewal through Laminin 511 matrix (Chang et al., 2015).

Other molecules can regulate BCSCs maintenance through Hippo/YAP/TAZ signaling. A recent study identify that FOXM1 is overexpressed in breast cancer and promotes proliferation, migration, and stemness through the Hippo signaling pathway (Sun et al., 2020). Another study shows that RUN1/3 acts as a negative regulator of YAP signaling and inhibits migration and stemness in breast cancer (Kulkarni et al., 2018). Mir-520b is also overexpressed in BCSCs and promotes stemness through the Hippo signaling (Zhang et al., 2019). Mir-125a regulated Hippo signaling through LIFR and promote BCSCs (Nandy et al., 2015). LncRNA SOX21-AS1 is overexpressed in breast cancer and promotes BCSCs, proliferation, invasion, and migration through promoting YAP nuclear translocation.

### 2.6 TGF-β signaling pathway

The TGF-β (Transforming growth factor beta) signaling pathway is a complex cellular signaling network that plays an important role in a variety of normal and pathological processes. TGF-β represents a family of soluble proteins including TGF-β1, TGF-β2 and TGF-β3, BMPs (bone morphogenic proteins), activin, growth differentiation factors (GDFs), nodal, and the müllerian inhibiting substance (MIS), which act through type I and II transmembrane serine-threonine receptors (Tzavlaki and Moustakas, 2020; Babyshkina et al., 2021).

The TGF-β signaling consists of two branches, a canonical pathway transduced via SMAD and a non-canonical pathway independent of SMAD proteins. These branches activate distinct target genes and frequently exhibit opposite functional roles. In canonical signaling, binding of TGF-β ligands induces the formation of a heterotetrameric active receptor complex (formed by a dimer of TGF-β and homodimers of both TGF-βRII and TGF-βRI) which results in the phosphorylation of TGF-βR1 by TGF-βR2. TGF-βI phosphorylates R-Smad proteins (Smad1/2/3/5/8), which form complexes with the common partner Smad (co-Smad; Smad4) and translocates to the nucleus to regulate the transcription of their target genes in conjunction with other DNA-binding transcription. Inhibitory SMADs such as SMAD6 and 7 (I-SMAD) can inhibit the signaling (Hata and Chen, 2016; Tzavlaki and Moustakas, 2020). In the non-canonical signaling also termed non-Smad pathways, TGF-β receptor complex phosphorylate alternative molecules such as TGF-β activated kinase 1 (TAK1), aPKC, Par6, Akt, and PI3K, which regulates several processes including apoptosis, proliferation, differentiation and migration (Zhang, 2017).

It is well known that TGF-β signaling is an important regulator of tumorigeneses by inducing epithelial-mesenchymal transition and regulating BCSCs maintenance. TGF-β ser 69 and 74 phosphorylation recruit SMAD3/p53 complex and regulate the transcription of BCSCs resistance genes (Zakharchenko et al., 2013). Zheng et al., (2014) showed that TGF-β2 expression correlates with the BCSCs marker ALDH1 and represents a bad prognosis. Another study showed that CD49F^high^ and CD61^high^ BCSCs are regulated by TGF-β in HER2+ breast cancer. (Lo et al., 2012). Recent studies have shown that TGF-β is capable to induce epithelial-mesenchymal transition, invasion, and lung metastasis and regulates apoptosis and resistance of BCSCs (Konge et al., 2018; Xu et al., 2018; Zhang et al., 2018; Katsuno et al., 2019; Yadav and Shankar, 2019; Tsubakihara et al., 2022).

Different molecules can regulate BCSCs maintenance through TGF-β. Iwanaga et al., show that Six1 promotes BCSCs through TGF-β and MAPK in luminal breast cancer (Iwanaga et al., 2012). In claudin-low breast cancer, NEDD9 is necessary to promote the expansion of BCSCs mediated by TGFβ/Smad and Rho-actin-SRF-dependent signals (Bruna et al., 2012).

A recent study showed that PARP3 promotes stemness and TGFβ-dependent EMT by inducing the Snail-E-cadherin axis and facilitating the acquisition of cell motility (Karicheva et al., 2016). Recent evidence indicates that RAD18 promotes the stem-cell phenotype through the Hippo/YAP/TGF-β pathway, resulting in the activation of M2 tumor-associated macrophages in triple-negative breast cancer (Yan et al., 2022). In addition, macrophages are capable to induce ERK/TGF-β1 signaling and promote BCSCs (Kundu and Shankar, 2022). Histamine H4 agonists also promote BCSCs and epithelial-mesenchymal transition through TGF-β and Src signaling (Galarza et al., 2020). Aurora-A kinase mediates TGF-β activation and promotes ALDH-positive cells, self-renewal, and resistance in breast cancer (Jalalirad et al., 2021). ALG3 promotes radioresistance and stemness through TGF-βRII glycosylation (Sun et al., 2021) ILEI/LIFR regulates BCSCS through TGF-β (Woosley et al., 2019). It has been observed that Leptin 1 and COX2 promote BCSCs through TGF-β1 (Mishra et al., 2017; Tian et al., 2017).

It has been reported that Autophagy promotes BCSCs through STAT3 and TGF-β/SMAD signaling (Yeo et al., 2016). Hyaluronan promotes aggressiveness in tumors through induce BCSCs, EMT, snail, twist, and TGF-β signaling (Chanmee et al., 2014). TGF-β overexpresses PMEPA1 and increases BCSCs (Nie et al., 2015).

## 3 Targeting signaling pathways as therapeutic strategies for breast cancer stem cells

Since, several signaling pathways regulating CSCs are known to be dysregulated in CSCs and contribute to CSCs survival, inhibitors of these pathways have been developed and tested in preclinical and clinical studies. Targeting signaling pathways regulating CSCs is a promising therapeutic approach for cancer treatment. Various authors have evaluated the effect of signaling pathway inhibitors in BCSCs as an alternative therapeutic approach for treating breast cancer (Table 1).

**TABLE 1:** Molecular targets to reduce BCSCs by targeting signaling pathways.

Molecule	Signaling pathway	Target	Effect	References
Anti-Fzd7 (SHH002-hu1)	Wnt signaling	Frizzled-7	Reduces bevacizumab-induced proliferation, migration, invasion, and epithelial-mesenchymal transition of triple-negative breast cancer cells by blocking CTC self-renewal	Xie et al. (2021)
Apatinib	Wnt signaling	RTK inhibitor	It reduces cell viability, migration, invasion, clonogenic capacity, sphere formation capacity and blocks the expression of markers associated with the stem phenotype of breast cancer cell lines	Jiang et al. (2022)
CWP232228	Wnt signaling	β-catenin/TCF	Reduces the clonogenic self-renewal capacity of CTCs. It also prevents the resistance of breast cancer cell lines to conventional chemotherapeutic treatments	Jang et al. (2015a)
Diallyl Trisulfide	Wnt signaling	-	It reduces the ability to mammospheres formation, decreases the expression of stem-associated markers, inhibits cell proliferation, and induces apoptosis in breast cancer cell lines	Li et al. (2018a)
Diosgenin	Wnt signaling	-	It inhibits cell proliferation and induces apoptosis of CTCs by favoring the expression of the Wnt antagonist sFRP4 from breast cancer cell lines	Bhuvanalakshmi et al. (2017)
Hydroxytyrosol	Wnt signaling	-	It reduces the population of CTC CD44+/CD24- and ALDH positive. It decreases epithelial-msenchymal transition, migration and invasion of breast cancer cell lines	Cruz-Lozano et al. (2019)
LGK-974	Wnt signaling	**PORCN**	It reduces carboplatin resistance and the expression of genes associated with the stem phenotype of breast cancer cell lines	Abreu de Oliveira et al. (2021)
Oxymatrine	Wnt signaling	-	Decreases of side population cells in breast cancer cell lines	Zhang et al. (2011)
PKF118–310	Wnt signaling	TCF4	Reduces the expression of epithelial-mesenchymal transition and stemness markers in combination with the inhibition of SAHA histone deacetylases. In addition, it reduces tumor growth in breast cancer cells	Hallett et al. (2012), Shamsian et al. (2020)
Plumbagin	Wnt signaling	-	It inhibits clonogenic capacity and the expression of markers associated with stemness, tumorigenesis, and metastasis of breast cancer cells	Sakunrangsit and Ketchart (2019)
Prodigiosin	Wnt signaling	-	Inhibits proliferation, migration, and tumorigenesis in breast cancer cell lines	Wang et al. (2016)
Resveratrol	Wnt signaling	-	Decreases CTC *in vivo* and *in vitro* of breast cancer cell lines	Fu et al. (2014)
Ursolic acid	Wnt signaling	-	It reduces stem characteristics by overexpressing the Wnt inhibitor sFRP4 and suppressing the expression of miR-499a-5p in breast cancer cell lines	Mandal et al. (2021)
6-Methoxymellein	NF-κB signaling	-	It inhibits cell proliferation and migration, reduces the CD44+/CD24- population, decreases the ability to form mammospheres and the expression of markers associated with the stem phenotype of breast cancer cell lines	Liu et al. (2020a)
Anthocyanins	NF-κB signaling	-	It inhibits cell proliferation and avoids cell resistance to conventional chemotherapeutic treatments of breast cancer cell lines	Paramanantham et al. (2020)
Aspirin	NF-κB signaling	-	It avoids chemoresistance of breast cancer cells by reducing the acquisition of the stemness of these cells	Saha et al. (2016)
Disulfiram	NF-κB signaling	-	Reduces stem characteristics and induces paclitaxel-mediated cytotoxicity of breast cancer cell lines	Yip et al. (2011)
Eugenol	NF-κB signaling	-	It prevents cisplatin resistance of breast cancer cell lines by blocking the expansion of ALDH-positive CTCs	Islam et al. (2018)
Machilin D	NF-κB signaling	-	It inhibits cell migration and invasion, as well as stem characteristics of breast cancer cell lines	Zhen et al. (2020)
Nalbuphine	NF-κB signaling	-	It inhibits cell proliferation, several stem characteristics, the epithelial-mesenchymal transition, and tumorigenesis of breast cancer cell lines	Yu et al. (2019)
Pterostilbene	NF-κB signaling	-	It inhibits the acquisition of stem cell characteristics and the metastatic potential induced by M2 macrophages of breast cancer cells	Mak et al. (2013)
Sulconazole	NF-κB signaling	-	It inhibits proliferation, tumor growth, mammospheres formation and the expression of stem markers of breast cancer cell lines	Choi et al. (2019)
Sulforaphane	NF-κB signaling	-	Blocks CTC expansion and sensitizes cells to chemotherapy in breast cancer cells	Burnett et al. (2017)
Tanshinone IIA	NF-κB signaling	-	It blocks several stem cell features by inhibiting IL-6/STAT3/NF-kB signaling in breast cancer cells	Lin et al. (2013)
Celastrol	Notch signaling	-	Reduces the ability to mammospheres formation and the expression of markers associated with the stemness of triple negative breast cancer cell lines	Ramamoorthy et al. (2021)
DAPT	Notch signaling	γ-secretase	Reduces stemness characteristics, the expression of markers associated with stem *in vitro*, as well as metastasis and tumorigenesis *in vivo*	McGowan et al. (2011), Farnie et al. (2013)
GSIXII	Notch signaling	γ-secretase	It induces apoptosis and reduces the mammosphere-forming capacity of breast cancer cell lines	Séveno et al. (2012)
hN1-NRR/Fc (Anti-Notch antiboy)	Notch signaling	Notch	Inhibits tumorigenesis and decreases stemness *in vivo* and *in vitro* of breast cancer cell lines	Qiu et al. (2013)
LY-411575	Notch signaling	γ-secretase	Decreases the ability to form mammospheres and the ability to form colonies on soft agar of breast cancer cell lines	Grudzien et al. (2010)
MRK-003	Notch signaling	γ-secretase	It decreases the ability to form mammospheres, the ability to form colonies on soft agar, and tumorigenesis in breast cancer cells	Grudzien et al. (2010), Kondratyev et al. (2012)
Notch-1-Fc	Notch signaling	Notch	Decreases proliferation and the ability to form mammospheres of breast cancer cell lines	Grudzien et al. (2010)
Psoralidin	Notch signaling	-	Reduces the population of ALDH-positive CTCs, epithelial-mesenchymal transition, and tumorigenesis of breast cancer cell lines	Pal et al. (2017)
Triptolide	Notch signaling	-	Reduces the expression of stemness-associated markers, and formation of mammospheres in triple-negative breast cancer cell lines	Ramamoorthy et al. (2021)
Z-Leu-Leu-Nle-CHO	Notch signaling	γ-secretase	Decreases the ability to form colonies on soft agar and mammospheres of breast cancer cell lines	Grudzien et al. (2010)
Curcumin	Wnt and Hedgehog signaling	-	It decreases the ability to form mammospheres, the expression of markers associated with stemness, and induces CTC apoptosis of breast cancer cell lines	Li et al. (2018b)
GANT61	Hedgehog signaling	Gli	It decreases cell proliferation, increases apoptosis and decreases the ability to form mammospheres of ER + breast cancer cell lines	Kurebayashi et al. (2017)
Genistein	Hedgehog signaling	-	Decreases cell proliferation, CTC ratio and tumorigenicity of breast cancer cell lines	Fan et al. (2013)
HPI-1	Hedgehog signaling	Gli	Decreases proliferation, migration and stemming of breast cancer cell lines	Jeng et al. (2018)
Huaier aqueous extract	Hedgehog signaling	-	It decreases cell viability, the ability to form mammospheres and the CD44^+^ CD24^−^ CTC population of breast cancer cell lines	Wang et al. (2014b)
Metformin	Hedgehog signaling	-	Decreases proliferation, migration, metastasis, tumorigenesis and stemming of breast cancer cell lines	Fan et al. (2015)
Nitidine Chloride	Hedgehog signaling	-	It decreases cell viability, cell migration, the expression of epithelial-mesenchymal transition genes and the stemness of breast cancer cell lines	Sun et al. (2016)
Salinomycin	Hedgehog signaling	-	Decreases cell proliferation, increases apoptosis, decreases migration, and stemness of breast cancer cell lines	Lu et al. (2015)
Thiostrepton	Hedgehog signaling	-	Reduces proliferation, self-renewal, and the expression of markers associated with stemness of breast cancer cells	Yang et al. (2016)
Physalin A	Hedgehog and YAP/TAZ signaling		It inhibits cell proliferation, the ability to form mammospheres, the expression of markers associated with stemness and the CD44^+^ CD24^−^ and ALDH-positive CTC population of breast cancer cell lines	Ko et al. (2021)
Chlorpromazine	YAP/TAZ signaling	-	Reduces the ability to mammospheres forming, the expression of markers associated with the stem phenotype and the resistance to chemotherapies of breast cancer cell lines	Yang et al. (2019)
Ciclesonide	YAP/TAZ signaling	-	It reduces proliferation, tumorigenesis, mammospheres formation capacity, and stemness markers through the glucocorticoid receptor-dependent YAP signal	Kim et al. (2020)
Quinacrine	YAP/TAZ signaling	-	Reduces the expression levels of molecules of the YAP/TAZ pathway in CTC of breast cell lines	Darbankhales et al. (2020)
Verteporfin	YAP/TAZ signaling	YAP	It decreases the expression of genes associated with the stem phenotype, cell viability, and resistance to chemotherapy in breast cancer cell lines	Guimei et al. (2020)
EW-7197	TGF-β signaling	ALK5	Decreases tumor growth *in vivo*, epithelial-mesenchymal transition, and paclitaxel-induced truncal characteristics in breast cancer	Park et al. (2015)
LY2157299	TGF-β signaling	TβRI	It reduces tumorigenesis by inhibiting stem characteristics of breast cancer cell lines	Bhola et al. (2013)
Vactosertib	TGF-β signaling	TβRI	It blocks the increase in cell migration, epithelial-mesenchymal transition, stemness, and the increase in reactive oxygen species caused by radiation. In addition, it inhibits lung metastasis *in vivo* from breast cancer cell lines	Choi et al. (2022)
ZL170	TGF-β signaling	TGFß/BMP	Reduces migration, invasion, proliferation, epithelial-mesenchymal transition, and stem characteristics *in vitro*. In addition, it reduces tumorigenesis and metastasis to bone and lung *in vivo* of breast cancer cells	Di et al. (2019)

Accumulated evidence has shown that Wnt/β-catenin inhibition decreases BCSCs, metastasis, and resistance to conventional therapy (Jang et al., 2015b; Shamsian et al., 2020; Abreu de Oliveira et al., 2021). A study in 2021 shows that antibodies against Frizzled-7 are capable of reducing invasion, migration, epithelial-mesenchymal transition, and breast cancer stem cell fraction in triple-negative breast cancer (Xie et al., 2021). Another study showed that apatinib is capable of reducing BCSC through Wnt/β-catenin inhibition. (Jiang et al., 2022). In the same way, the small molecule inhibitors PKF118–310 and CWP23228 decrease BCSCs by disrupting the interaction between β-catenin and Tcf/Lef transcription factors, thereby inhibiting Wnt target gene expression (Hallett et al., 2012; Jang et al., 2015a).

Furthermore, organic components such as diallyl trisulfide, ursolic acid, curcumin, plumbagin, prodigiosin, diosgenin, hydroxytyrosol, resveratrol, and oxymatrine are capable of inhibiting Wnt/β-catenin-induced BCSCs (Zhang et al., 2011; Fu et al., 2014; Wang et al., 2016; Bhuvanalakshmi et al., 2017; Li et al., 2018a, 2018b; Cruz-Lozano et al., 2019; Sakunrangsit and Ketchart, 2019; Mandal et al., 2021).

It is also well known that NF-κB inhibition impairs the self-renewal of BCSCs. A study in 2019 shows that nalbuphine is capable to inhibit AKT/NF-κB signaling and reduce BCSCs (Yu et al., 2019). Organic components such as 6-methoxymellein, anthocyanins, pterostilbene, eugenol, tanshinone IIA, sulforaphane, and machilin D are capable to inhibit NF-κB-induced BCSCs and drug resistance in breast cancer (Lin et al., 2013; Mak et al., 2013; Burnett et al., 2017; Islam et al., 2018; Liu R. et al., 2020; Paramanantham et al., 2020; Zhen et al., 2020). Furthermore, disulfiram and aspirin disrupt the NF-κB signaling by inhibiting the phosphorylation and degradation of IκBα (Yip et al., 2011; Saha et al., 2016). Sulconazole, also inhibits the translocation of NF-κB from cytoplasm to the nucleus, thus disrupting the NF-κB pathway. (Kastrati et al., 2017; Choi et al., 2019). Parthenolide, pyrrolidine dithiocarbamate, and its analog diethyldithiocarbamate were found to inhibit the NF-kB activity and diminish the proliferation and colony formation of BCSCs (Zhou et al., 2008). *In vivo*, PDTC was also able to disrupt tumor onset and growth, and its effect was enhanced by paclitaxel (Zhou et al., 2008).

Disruption of the notch signaling reduces BCSCs, resistance, and metastasis *in vivo* and *in vitro* in breast cancer. (Grudzien et al., 2010; McGowan et al., 2011; Simmons et al., 2012; Qiu et al., 2013; Peng et al., 2014). γ-secretase inhibitors reduce the CSCs population and inhibit self-renewal. A study shows that γ-secretase inhibitors in combination with ErbB1/2 inhibitors (lapatinib and gefitinib) decrease CSCs in preclinical models (Farnie et al., 2013). Other γ-secretase inhibitors such as MRK-003 and GSIXII inactivate Notch signaling and decrease BCSCs in mice (Kondratyev et al., 2012; Séveno et al., 2012). Furthermore, natural components such as psoralidin, celastrol y triptolide are capable to inhibit BCSCs and epithelial-mesenchymal transition by disrupting the Notch signaling pathway. (Pal et al., 2017; Ramamoorthy et al., 2021).

It is well demonstrated that Hedgehog inhibition reduces the BCSCs fraction, impairs self-renewal, and sensitizes CSCs to drug therapy. Gli1/2 inhibitors such as GANT61 and HPI-1 reduce proliferation, and migration, increase apoptosis and reduce BCSCs (Kurebayashi et al., 2017; Jeng et al., 2018). Furthermore, natural compounds such as nitidine chloride, huaier aqueous extract, physalin A, and genistein are capable to impaired migration, and invasion and reducing BCSCs through Hedgehog inhibition (Fan et al., 2013; Wang X. et al., 2014; Sun et al., 2016; Ko et al., 2021). Pharmacological components such as metformin, salinomycin, and thiostrepton inhibit Hedgehog signaling and the BCSCs population (Fan et al., 2015; Lu et al., 2015; Yang et al., 2016). In addition, many studies have shown that the inhibition of YAP, a transcription factor regulating BCSCs, reduces drug resistance in breast cancer (Guimei et al., 2020). Further, pharmacological components such as quinacrine are capable to reduce YAP and LATS1/2 expression in breast cancer (Darbankhales et al., 2020). Chlorpromazine inhibits YAP and decreases BCSCs resistance (Yang et al., 2019). Ciclesonide is capable to inhibit glucocorticoid receptor-dependent YAP signaling and decreasing BCSCs (Kim et al., 2020). The natural component physalin A also reduces YAP1 levels in breast cancer and impacts the proportion of BCSCs (Ko et al., 2021).

TGF-β inhibition may target CSCs by promoting their differentiation and blocking their expansion (Sulaiman et al., 2021). TGF-β inhibition mediated by LY2157299, an inhibitor of the TGF-β receptor 1 kinase, and a siRNA against SMAD4 blocks BCSCs expansion, impairs mammosphere formation and reduces drug resistance (Bhola et al., 2013). Vactosertib targets the kinase activity of ALK5 (Activin receptor-like kinase 5), which is a receptor for the TGF-β signaling pathway. By inhibiting ALK5, vactosertib can interfere with TGF-β signaling and reduces epithelial-mesenchymal transition, *in vivo* metastasis*,* and BCSC fraction through ROS reduction (Choi et al., 2022). ALK5 and EW-7197 inhibitors reduce paclitaxel-promoting epithelial-mesenchymal transition and BCSC numbers, suggesting that combined treatment of paclitaxel with TGF-β inhibitors attenuate breast cancer metastasis and BCSCs (Park et al., 2015). ZL170 inhibits proliferation, epithelial-mesenchymal transition, stemness, invasion, and migration via inhibition of TGF-β/BMP-SMAD pathways in triple-negative breast cancer (Di et al., 2019). Caffeic acid reduces BCSCs for induction of microRNA-148a. These microRNAs inhibit TGF-β/SAMD2 signaling (Li et al., 2015).

Collectively these data depicted inhibitors of signaling pathways showing promise for the treatment of breast cancer, and several agents are currently being tested either as monotherapy or in combination with other conventional therapies. Overall, targeting signaling pathways regulating CSCs is a promising therapeutic approach for cancer treatment, but further research is needed to overcome the challenges and optimize the clinical efficacy of this approach.

## 4 Conclusion

BCSCs are a group of cells within tumors with the ability to self-renew and differentiate into non-CSCs that form the bulk of the tumor. CSCs are responsible for tumor maintenance, resistance, and relapse in breast cancer patients. Targeting CSCs has emerged as a promising therapeutic approach for the treatment of cancer. In recent years, significant progress has been made in developing therapeutic interventions for targeting CSCs. These interventions include the use of small molecules to inhibit the signaling pathways that are esential for the maintenance and self-renewal of CSCs, such as the Wnt, NF-kB, Notch, Hedgehog, Hippo, and TGF-β signaling pathways. Recent approaches have revealed that the inhibition of key molecules involved in those signaling pathways reduces the CSC fraction and impairs self-renewal.

Although significant advancements have been achieved, there are still several challenges that need to be addressed. First, CSCs are heterogeneous and could rely on different signaling pathways depending on the tumor type and stage. Second, CSCs may adapt to the inhibition of a given single pathway by activating compensatory pathways. Third, some signaling pathways also play important roles in normal stem cells and tissues, which can lead to toxic side effects. Therefore, combination therapies targeting multiple pathways or combining CSC-targeting agents with conventional chemotherapy or radiotherapy could be more effective in eliminating CSCs and preventing tumor recurrence.

In conclusion, the development of CSC-targeted therapies represents an exciting area of research with the potential to revolutionize cancer treatment. Despite the challenges, progress has been made in developing therapeutic interventions that can effectively target CSCs. Continued research in this field is crucial for creating more effective and specific CSC-targeted therapies that can ultimately improve patient outcomes.
